# Analysis of Calculated Liver Scores for Long-Term Outcome in 423 Cutaneous Melanoma Patients

**DOI:** 10.3390/cancers16183217

**Published:** 2024-09-21

**Authors:** Nessr Abu Rached, Mariana Marques da Silva Reis, Eggert Stockfleth, Riina Käpynen, Thilo Gambichler

**Affiliations:** 1 Skin Cancer Center, Department of Dermatology, Venereology and Allergology, Ruhr-University Bochum, 44791 Bochum, Germany; mariana.reis@kklbo.de (M.M.d.S.R.); eggert.stockfleth@klinikum-bochum.de (E.S.); riina.kaepynen@kklbo.de (R.K.); thilo.gambichler@kklbo.de (T.G.); 2 Department of Dermatology and Phlebology, Christian Hospital Unna, 59423 Unna, Germany; 3 Department of Dermatology, Faculty of Health, School of Medicine, Dortmund Hospital, Witten/Herdecke University, 58455 Dortmund, Germany

**Keywords:** APRI, aspartate transaminase-to-platelet ratio index, melanoma, prognosis, skin cancer, cutaneous melanoma

## Abstract

**Simple Summary:**

Cutaneous melanoma (CM) is an aggressive skin cancer that develops from melanocytic cells. The most important prognostic factor is still the vertical tumour thickness according to Breslow. Liver function is associated with better overall patient outcomes and is currently under increasing investigation in cancer patients. Current findings show that liver function is an important factor in different tumour entities and represents a potential biomarker. Therefore, we aimed to evaluate liver metabolism by using liver scores in CM. High tumour thickness (≥1.66 mm) and aspartate transaminase-to-platelet ratio index (APRI ≥ 0.2241) at the initial diagnosis were associated with a worse prognosis in stage I and II melanoma.

**Abstract:**

**Background:** Neoadjuvant and adjuvant therapies are currently getting increasingly important in cutaneous melanoma (CM) management. However, there is still a lack of prognostic tools to identify which patients have a poor prognosis. There is increasing evidence that the liver score may be a potential prognostic parameter in different tumour types. The aim was to investigate whether established liver scores can establish the prognosis of CM. **Methods:** According to established methods, the APRI, the MELD score, the MELD-Na score and the De Ritis ratio were calculated from the laboratory values at the time of the initial diagnosis. Survival was compared with the Kaplan–Meier curve and tested with log-rank tests. Risk factors associated with cutaneous melanoma-specific survival (CMSS) and progression-free survival (PFS) were assessed by using the Cox proportional hazards regression model. To determine the diagnostic accuracy, we performed a time-dependent ROC analysis. Results: A total of 423 patients were included, including 141 patients in AJCC stage (2017) I (33.3%), 82 in stage II (19.4%), 128 in stage III (30.3%) and 72 in stage IV (17%). Median time until melanoma-specific death was 99 months (IQR: 37–126). In addition, 37.6% of patients relapsed with a median time to relapse of 88 months (IQR: 17.5–126). In all stages, tumour thickness and ulceration were independent markers for predicting CMSS and PFS (*p* < 0.05). The multivariable analysis with all stages showed no significant association with CM outcome for liver scores (*p* > 0.05). The subgroup analysis revealed that the APRI (≥0.2241) was associated with CMSS and PFS in melanoma stages I and II, independently of tumour thickness, age and ulceration (HR 2.57, 95% CI 1.14–5.75; HR 2.94, 95% CI 1.42–6.09, respectively). **Conclusions:** The 20-year prognosis of AJCC stage I and II CM was dependent on tumour thickness and the APRI. High tumour thickness and an APRI ≥ 0.2241 at the initial diagnosis were associated with a worse prognosis. Future studies should investigate the independent prognostic value of the APRI in low-stage CM. Furthermore, the APRI score could be a potential biomarker for nomograms.

## 1. Introduction

Cutaneous melanoma (CM) is an aggressive skin cancer that develops from melanocytic cells. Known risk factors include high UV exposure, fair skin type, a large number of nevi and a positive family history of melanoma [1]. According to Breslow, the prognosis in the metastatic stage depends on the thickness of the tumour and has improved significantly with immune checkpoint inhibitors and targeted therapies [2,3]. Thin tumours typically have a good prognosis, but there are also tumours that relapse despite having a low tumour thickness (e.g., <1 mm). Prognostic markers at low tumour thickness are not well studied. Currently, neoadjuvant and adjuvant therapies in the early stages of CM are being discussed. For this reason, new biomarkers and additional other risk factors have recently been investigated. In Merkel cell carcinoma (MCC), there is evidence that liver scores may be helpful in estimating prognosis. In particular, the MELD (model for end-stage liver disease) score, which has long been used to assess mortality in cirrhosis, was found to predict MCC-specific death and relapse in MCC [4,5].

The liver is a vital organ in the human body and has several important functions that are crucial for health and survival. Some of its main functions include metabolism, detoxification, protein synthesis, vitamin storage, energy storage and hormone regulation. In addition, liver scores and liver function are indicators of a patient’s overall health [6]. Patients with good liver function tend to have better general health and therefore a better ability to tolerate and recover from intensive cancer treatments. Thus, it is possible that patients with tumours may show changes in the liver and liver function before radiological findings are detected. In addition, better liver function is associated with a better prognosis in tumour patients [7].

The APRI (aspartate transaminase-to-platelet ratio index) is a useful non-invasive marker for estimating the degree of liver fibrosis in patients with chronic liver disease [8]. The APRI score is calculated as the ratio of aspartate aminotransferase (AST) to the upper limit of the normal range divided by the number of platelets [9]. The MELD score is a tool developed to assess the severity of chronic liver disease and the urgency of liver transplantation [10,11]. This score is based on laboratory values that reflect liver and kidney function and coagulation status. In addition, a retrospective study in 39,323 hospitalised patients showed that the MELD score is an independent predictor of mortality [12]. The MELD-Na score is an advanced version of the MELD score that includes serum sodium concentration [13]. The De Ritis ratio is the ratio of AST to alanine aminotransferase (ALT) and is used to assess the type and severity of liver disease [14].

The use of established liver scores for predicting CM have not been investigated so far. To fill this gap, we aimed to systematically evaluate several established liver scores, including, for the first time, the APRI, the MELD score, the MELD-Na score and the De Ritis ratio (ratio of aspartate aminotransferase to alanine aminotransferase), with CM outcomes (cutaneous melanoma-specific survival [CMSS] and progression-free survival [PFS]) in a large monocentric, retrospective study. In particular, we aimed to investigate whether the different liver scores calculated at the time of the initial diagnosis could be useful.

## 2. Materials and Methods

### 2.1. Design and Setting

In this monocentric study, we retrospectively analysed the laboratory, demographic and clinical data of 423 patients with melanoma who were treated at Skin Cancer Center Bochum. CM patients in all AJCC 2017 (8th edition) stages who were over 18 years at the initial diagnosis were included. Patients were excluded if they had other malignancies at the time of diagnosis or had incomplete laboratory data. Demographic data (including gender, age at diagnosis and Charlson comorbidity index [CCI]), disease-specific data (including tumour thickness and presence of ulceration), and laboratory and survival data (including CMSS and PFS) were extracted from the patient records. This study was conducted in accordance with the ethical principles of the Declaration of Helsinki [15]. This study was approved by the Institutional Ethics Review Board of Ruhr University Bochum (ethics vote: #16-5985).

### 2.2. Patients and Data Collection

A total number of 423 consecutive CM patients treated at our centre was included in this study. All patients with complete demographic, laboratory and disease-specific data were included. The laboratory parameters collected included bilirubin, creatinine, sodium, international normalised ratio (INR), ALT, AST and platelet count. The liver scores APRI, MELD score, MELD-Na score [16] and De Ritis ratio were calculated as follows:

$$MELD\;score=3.78\times ln\left(serum\;bilirubin\left[\frac{mg}{dL}\right]\right)+11.2\times ln\left(INR\right)+\;9.57\times ln\left(serum\;creatinine\left[\frac{mg}{dL}\right]\right)+6.43,$$

$$MELD-NA\;score=MELD\;score-Na\left[\frac{mmol}{l}\right]-(0.025\times MELD\times \left(140-Na\left[\frac{mmol}{l}\right]\right))+140,$$

$$APRI=\left(aspartate\;aminotransferase\left[\frac{U}{l}\right]-\;upper\;reference\;value\;of\;aspartate\;aminotransferase\left[\frac{U}{l}\right]\right):platelet\;count[\frac{10^{9}}{l}]$$

$$De\;Ritis\;ratio=aspartate\;aminotransferase\left[\frac{U}{l}\right]:alanine\;transaminase\left[\frac{U}{l}\right].$$

Univariable and multivariable analyses were performed to investigate the role of liver scores in predicting outcome. Patients were treated according to the German CM guidelines [17]. To characterise the mortality risk of comorbidities, the CCI was evaluated. The latter was calculated based on age and comorbidities (history of myocardial infarction, chronic heart failure, hemiplegia, dementia, chronic kidney disease, cerebrovascular accident/TIA, connective tissue disease, peptic ulcer disease, COPD, liver disease, diabetes mellitus, leukaemia, lymphoma, solid tumour and AIDS).

### 2.3. Data Analysis

Normally distributed continuous variables were expressed as means ± standard deviations, and non-normally distributed continuous variables were expressed as medians and interquartile ranges. For categorical variables, percentages and frequencies were used. A non-parametric test (Mann–Whitney U test) was used to detect differences between two groups of continuous variables. The chi-squared test was used to investigate differences between categorical variables.

Survival analysis for PFS and CMSS was performed by using the Kaplan–Meier method and compared by using the log-rank test. For multivariable analysis, a Cox proportional hazards regression model was used to estimate the association between different variables and PFS or CMSS. Variables with log-rank test *p* < 0.05 in the Kaplan–Meier survival analysis were used to construct the Cox proportional hazards regression model. To determine the cut-off value and diagnostic accuracy, a time-dependent ROC analysis with determination of the area under the curve (AUC) was conducted. The statistical analyses were carried out by using IBM SPSS Statistics version 29.0 (IBM Corporation, New York, NY, USA). The statistically significant level was set to *p* < 0.05.

## 3. Results

### 3.1. Patient and Cutaneous Melanoma Characteristics

After reviewing inclusion and exclusion criteria, a total of 423 unselected cases of melanoma with pathological confirmation were included in this retrospective study (Table 1). Sex distribution was balanced, with 213 patients (50.4%) being male and 210 female (49.6%). The interquartile range (ICR) of age at diagnosis ranged from 46 to 70 years (median of 59 years). There were 141 patients in AJCC stage (2016) I (33.3%), 82 in stage II (19.4%), 128 in stage III (30.3%) and 72 in stage IV (17%).

Table 2 presents the clinical data of the patients in AJCC stages I and II. The median tumour thickness in AJCC I and II was 1.3 mm (range 0.18–10). About a quarter of AJCC stage I and II patients had ulceration (24.7%, n = 55). The liver scores included the median MELD score (6.7, ICR 6.4–7.3), the MELD Na score (9.3, ICR 9–10), the APRI score (median 0.24, ICR 0.18–0.33) and the De Ritis ratio (median 1.2, ICR 0.9–1.4). The outcome data included CMSS and PFS. CM-specific death occurred in 15.3% of AJCC stage I and II patients (n = 34), with a median survival of 113 months (IQR 87–132). In addition, CM relapse was observed in 20.6% of AJCC stage I and II patients (n = 46), with a median relapse time of 113 months (IQR 84.5–131).

### 3.2. Univariable and Multivariable Analyses of CM Patients with AJCC I to IV (All Patients)

We performed univariable and multivariable analyses to determine the relationship between the different liver scores and all CM patients (AJCC I to IV). In the univariable analysis, CM-specific death was associated with tumour thickness, the CCI score, age at the first diagnosis, the MELD score, the MELD-Na score and the presence of ulceration (*p* < 0.001, <0.001, <0.001, 0.041, 0.007 and <0.001, respectively). CM relapse was also related to tumour thickness, the CCI score, age at the first diagnosis, the MELD score, the MELD-Na score and the presence of ulceration (*p* < 0.001, <0.001, <0.001, 0.049, 0.009 and <0.001, respectively). The APRI score, sex and the De Ritis quotient of the entire CM group in AJCC I to IV were not associated with CM-specific death and CM relapse (*p* > 0.05). The univariable survival analysis using the Kaplan–Meier method showed an association between CMSS and the MELD-Na score (*p* = 0.011), age (*p* < 0.001), tumour thickness (*p* < 0.001) and the presence of ulceration (*p* < 0.001). Associations were also seen between PFS and the MELD-Na score (*p* = 0.023), age (*p* < 0.001), tumour thickness (*p* < 0.001) and the presence of ulceration (*p* < 0.001). The MELD score, the APRI, the De Ritis quotient and sex were not statistically significant with respect to CMSS and PFS (*p* > 0.05).

Variables with log-rank test *p* < 0.05 in the Kaplan–Meier survival analysis (MELD-Na score, age, tumour thickness and presence of ulceration) were used to construct the Cox proportional hazards regression model. In the Cox proportional hazards regression model (overall model, *p* < 0.001), only age (HR 1.03; 95% CI 1.02–1.05; *p* < 0.001 and HR 1.09; 95% CI 1.04–1.14; *p* < 0.001) and tumour thickness (HR 1.16; 95% CI 1.09–1.23; *p* < 0.001 and HR 1.3; 95% CI 1.13–1.45; *p* < 0.001) remained significantly associated with CMSS and PFS. Ulceration (HR 2.52; 95% CI 1.65–1.85; *p* < 0.001) was associated with CMSS but not with PFS (HR 2; 95% CI 0.76–5.31; *p* = 0.126). MELD-Na was not significantly associated with CMSS or PFS in the model.

### 3.3. Univariable and Multivariable Analyses of CM Patients in AJCC I and II (Systemic Therapy-Naive Group at Initial Diagnosis)

As the outcome of CM is influenced by the different systemic therapies (immune checkpoint inhibitors, targeted therapy and chemotherapy), we performed a subgroup analysis in further analysis. This subgroup analysis included all patients who were not receiving systemic therapy at the time of initial diagnosis (all patients in AJCC stages I–II; no adjuvant therapy). In the univariable analysis of CM patients in AJCC stages I and II, MM-specific death was associated with tumour thickness, the CCI score, age at the first diagnosis, the APRI and the presence of ulceration (*p* < 0.001, 0.01, 0.014, 0.016 and 0.001, respectively). CM relapse was also associated with these variables (*p* < 0.001, 0.016, 0.038, 0.009 and 0.03, respectively). The MELD score, the MELD-Na score, the De Ritis quotient and sex showed no association with CM-specific death and CM relapse (*p* > 0.05). The univariable survival analysis using the Kaplan–Meier method showed an association between CMSS and the APRI (*p* = 0.009), age (*p* = 0.015), tumour thickness (*p* < 0.001), the CCI score (*p* = 0.017) and the presence of ulceration (*p* = 0.001). There was no correlation with PFS or CMSS for the other variables (MELD score, MELD-Na score, sex and De Ritis quotient; all *p* > 0.05).

**Figure 1:**
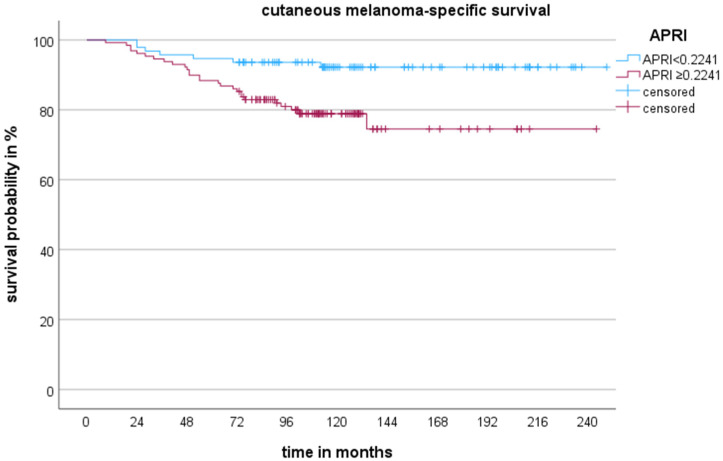
The 20-year survival probabilities are shown by Kaplan–Meier curves for patients with cutaneous melanoma in AJCC I and II. The curves show that an APRI ≥ 0.2241 was significantly associated with decreased cutaneous melanoma-specific survival (log-rank test: *p* = 0.001).

**Figure 2:**
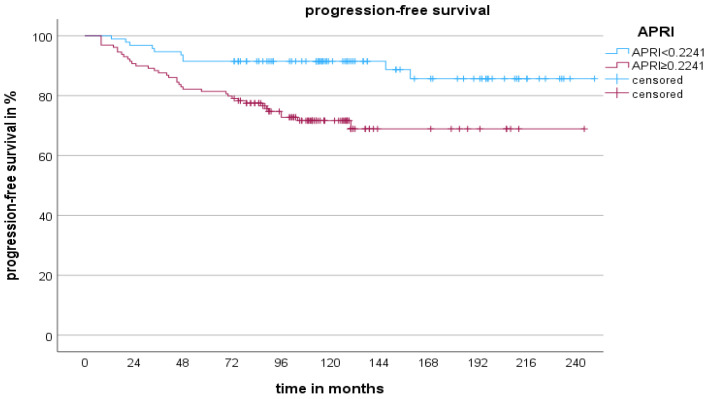
The 20-year progression-free survival rates are shown by Kaplan–Meier curves for patients with cutaneous melanoma in AJCC I and II. The curves show that an APRI ≥ 0.2241 was significantly associated with decreased progression-free survival rates (log-rank test: *p* = 0.001).

ROC analysis was used to determine the optimal cut-off values for age (≥52.5 years), tumour thickness (≥1.66 mm), the APRI (≥0.2241) and the CCI score (≥5.5). For the multivariable analysis, all variables with log-rank test *p* < 0.05 in the Kaplan–Meier survival analysis were used to construct the Cox proportional hazards regression model (age, tumour thickness, presence of ulceration, APRI and CCI score; Table 1 and Table 2). None of the patients had known liver disease or liver metastases, so this was not a confounding factor in our model. Table 3 presents the Cox proportional hazards regression model examining the risk factors associated with CM-specific death. The log-rank test indicates a highly significant result, with *p* < 0.001. Tumour thickness (≥1.66 mm), higher APRI scores (≥0.2241) and higher CCI scores (≥5.5) showed significant associations with increased risk of CM-specific death (hazard ratio [HR] 3.91, 95% confidence interval (CI) 1.81–8.45; HR 2.57, 95% CI 1.14–5.75; HR 2.93, 95% CI 1.24–6.89). Age and the presence of ulceration were not significant in the model for CMSS (*p* > 0.05). In our multivariable model, tumour thickness (≥1.66 mm) and higher APRI scores (≥0.2241) were associated with an increased risk of CM relapse (HR 4, 95% CI 2.1–7.63; HR 2.94, 95% CI 1.42–6.09; Table 4). There was no correlation between age, the presence of ulceration and the CCI score and PFS, respectively (*p* > 0.05). The Kaplan–Meier curves for PFS, CMSS and an APRI ≥ 0.2241 are shown in Figure 1 and Figure 2.

**Figure 3:**
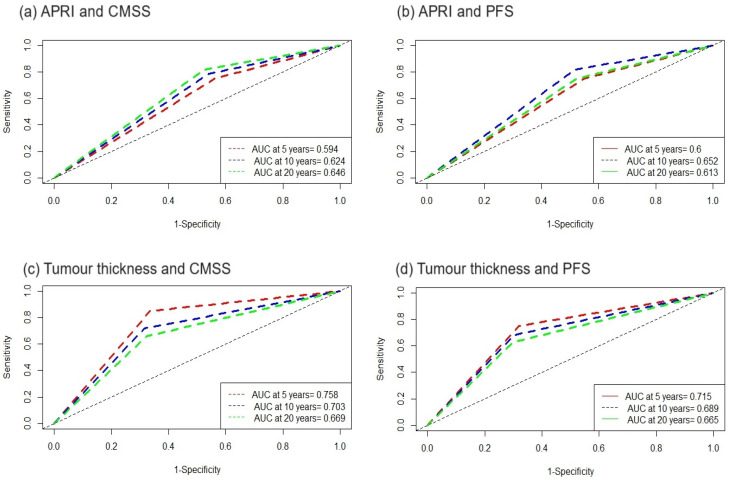
Time-dependent ROC analyses: (**a**) APRI ≥ 0.2241 in relation to cutaneous melanoma-specific survival (CMSS), (**b**) APRI ≥ 0.2241 in relation to progression-free survival (PFS), (**c**) tumour thickness ≥ 1.66 in relation to CMSS and (**d**) tumour thickness ≥ 1.66 in relation to PFS.

To determine the diagnostic accuracy, we performed a time-dependent ROC analysis. Figure 3 shows the results of the time-dependent ROC analysis. The area under the curve (AUC) for tumour thickness and CMSS or PFS at 5 years, 10 years and 20 years was higher than for the APRI. The difference in the AUC between tumour thickness and the APRI decreased over the years.

## 4. Discussion

Liver metastases are a common characteristic of advanced CM and determine the prognosis [18,19,20]. Between 14 and 20% of CM patients have clinically or radiologically detectable liver metastases [21]. In uveal melanoma, the rate is even around 50% of patients with liver metastases [22]. Contrast-enhanced MRI and contrast-enhanced CT are the first-line methods for detecting liver metastases, but both have limitations and may not be able to detect small micrometastases [23]. For example, pathological examinations of liver metastasectomies in patients with metastatic small intestinal or pancreatic tumours often revealed micrometastases that were not detectable by radiological or macroscopic gross examination [24]. The frequency of micrometastases in CM that are not detectable by radiological or macroscopic clinical examination is unknown. The early detection of liver metastases and the accurate assessment of their distribution are important for treatment management and CM patient prognosis. Liver scores (APRI, MELD score, MELD-Na score and De Ritis ratio) are calculated scoring systems based on various clinical and biochemical parameters and are used to assess liver function and health. The ability to use calculated liver scores as prognostic markers in CM may allow for a more accurate prediction of disease progression and the earlier detection of small, non-apparent micrometastases.

Elevated liver enzymes could be caused by non-apparent micrometastases in the liver [25,26]. For example, a retrospective study in breast cancer showed that AST, ALT and other liver values were significantly higher than normal 6 months before the detection of liver metastases [25]. It was also found that patients with breast cancer and liver metastases had significantly higher ALT, AST and other liver values than patients without liver metastases [27,28]. The De Ritis ratio was found to be of prognostic value in different types of cancer [29,30,31,32,33,34,35]. An association between tumour prognosis in neuroendocrine tumours and the De Ritis ratio has also been described [36]. A meta-analysis by Su et al. in 8565 patients with urological cancer also confirmed the impact of the De Ritis ratio in cancer [37]. To our knowledge, De Ritis ratio analysis was not performed in patients with CM. Our analysis also showed that there was no link between CM prognosis and the De Ritis ratio. In addition, the MELD liver score was associated with the prognosis in patients with Merkel cell carcinoma [4,5]. Furthermore, the MELD score at hospitalisation was significantly associated with mortality in a retrospective study of 39,323 inpatients [12]. We found no association between the MELD score and the prognosis in patients with CM. The APRI is a calculated parameter used to estimate the risk of liver fibrosis and cirrhosis in patients with chronic liver disease [38]. This score is calculated from aspartate aminotransferase (AST) levels and platelet counts in the blood and does not require a biopsy [38]. We showed for the first time that the APRI (≥0.2241) can predict CMSS and PFS in stage I and II melanoma, independently of tumour thickness, age and ulceration. Microinflammation in the liver, which is reflected in a higher APRI, could be caused by invisible liver metastases. On the other hand, this argument contradicts the fact that the APRI is not associated with stages III and IV. However, there may be a bias, as stage III and IV patients were treated with immunotherapy after the initial diagnosis. None of our patients received adjuvant immunotherapy in stage II. The reasons for the link between the liver and melanoma remain speculative. However, tumour thickness was associated with a higher diagnostic accuracy than the APRI. Tumour thickness was still the most accurate marker for establishing the prognosis in CM. Nonetheless, these results are important because nomograms are increasingly being used in oncology [39]. Oncological nomograms have a higher diagnostic accuracy than individual prognostic markers. Recent studies have developed nomograms for lung cancer, pancreatic cancer and breast cancer, among others [40,41,42]. Thus, the APRI is a possible component of a nomogram to establish the prognosis in CM.

A limitation of this study is its retrospective design. Potential undetected confounders may be a limitation of the retrospective design. Future studies should investigate the APRI in stage I and II melanoma. In addition, we recommend that the APRI be tested in a multi-centre setting. In addition, an independent dataset is required for the external validation of the APRI in CM.

## 5. Conclusions

In summary, this is the first study to investigate liver scores for their ability to establish the prognosis in CM. The APRI (≥0.2241) was found to be an independent marker associated with poorer outcome (CMSS and PFS) in melanoma stage I and II patients. Thus, the APRI may be suitable as a possible component of a nomogram for establishing the prognosis in CM.

## Figures and Tables

**Table 1:** All patient and cutaneous melanoma characteristics (n = 423).

Parameter		Value (s)
Sex, n (%)	Male	213 (50.4)
	Female	210 (49.6)
Age, median (ICR), y		59 (46–70)
AJCC 2017, n (%)	I	141 (33.3)
	II	82 (19.4)
	III	128 (30.3)
	IV	72 (17)
MELD score, median (ICR)		6.8 (6.4–7.5)
MELD Na score, median (ICR)		9.3 (8.9–10.2)
APRI score, median (ICR)		0.24 (0.18–0.33)
De Ritis ratio, median (ICR)		1.1 (0.86–1.33)
CCI score, median (ICR)		5 (3–7)
CM-specific death, n (%)	Yes	114 (27)
	No	309 (73)
CM death in months, median (IQR)		99 (37–126)
CM relapse, n (%)	Yes	159 (37.6)
	No	264 (72.4)
Relapse in months, median (IQR)		88 (17.5–126)

n, absolute number of patients; y, years; ICR, interquartile range; AJCC, American Joint Commission on Cancer; MELD, model for end-stage liver disease; APRI, aspartate transaminase-to-platelet ratio index; CCI, Charlson comorbidity index.

**Table 2:** Characteristics of melanoma patients in AJCC stages I and II (n = 223).

Parameter		Value (s)
Tumour thickness, median (range), mm		1.3 (0.18–10)
Tumour ulceration, n (%)	Yes	55 (24.7)
	No	168 (75.3)
pT, n (%)	T1a	21 (9.4)
	T1b	44 (19.7)
	T2a	77 (34.5)
	T2b	16 (7.2)
	T3a	33 (14.8)
	T3b	18 (8.1)
	T4a	2 (0.9)
	T4b	12 (5.4)
MELD score, median (ICR)		6.7 (6.4–7.3)
MELD-Na score, median (ICR)		9.3 (9–10)
APRI score, median (ICR)		0.24 (0.18–0.33)
De Ritis ratio, median (ICR)		1.2 (0.9–1.4)
CM-specific death, n (%)	Yes	34 (15.3)
	No	189 (84.8)
CM death in months, median (IQR)		113 (87–132)
CM relapse, n (%)	Yes	46 (20.6)
	No	177 (79.4)
Relapse in months, median (IQR)		113 (84.5–131)

pT, pathological stage; CM, cutaneous melanoma; ICR, interquartile range.

**Table 3:** Cox proportional-hazards regression model for CM-specific death (status: positive CM-specific death; time: time to death) including variables from the univariable Kaplan–Meier survival analysis with *p*-value ≤ 0.05 (n = 223); log-rank test *p* < 0.001.

Parameter	Hazard Ratio (HR)	95% Confidence Interval (CI)	*p*-Value
Tumour thickness (≥1.66 mm)	3.91	1.81–8.45	0.001 *
Presence of ulceration (yes)	1.69	0.83–3.44	0.15
Age (≥52.5 years)	1.64	0.66–4.04	0.28
APRI (≥0.2241)	2.57	1.14–5.75	0.022 *
CCI score (≥5.5)	2.93	1.24–6.89	0.014 *

CM, cutaneous melanoma; CCI score, Charlson comorbidity index; APRI score, aspartate aminotransferase to platelet count ratio index; * significant result.

**Table 4:** Cox proportional-hazards regression model for CM relapse (status: positive CM relapse; time: time to relapse) including variables from the univariable Kaplan–Meier survival analysis with *p*-value ≤ 0.05 (n = 223); log-rank test *p* < 0.001.

Parameters	Hazard Ratio (HR)	95% Confidence Interval (CI)	*p*-Value
Tumour thickness (≥1.66 mm)	4	2.1–7.63	<0.001 *
Presence of ulceration (yes)	1.14	0.61–2.16	0.68
Age (≥52.5 years)	1.45	0.71–2.95	0.31
APRI (≥0.2241)	2.94	1.42–6.09	0.004 *
CCI score (≥5.5)	1.9	0.8–4.07	0.097

CM, cutaneous melanoma; CCI score, Charlson comorbidity index; APRI score, aspartate aminotransferase to platelet count ratio index; * significant result.

## Data Availability

Derived data supporting the findings of this study are available from N.A.R. upon reasonable request.
